# Predictive Value of the Phase Angle for Analgesic Efficacy in Lumbosacral Transforaminal Block

**DOI:** 10.3390/jcm10020240

**Published:** 2021-01-12

**Authors:** Jeayoun Kim, Hue Jung Park, Woo Seog Sim, Seungwon Lee, Keoungah Kim, Woo Jin Kim, Jin Young Lee

**Affiliations:** 1 Department of Anesthesiology and Pain Medicine, Samsung Medical Center, Sungkyunkwan University, School of Medicine, Seoul 06351, Korea; jeayoun.kim@samsung.com (J.K.); wooseog.sim@samsung.com (W.S.S.); seungwon0209.lee@samsung.com (S.L.); keoungah.kim@samsung.com (K.K.); wj888.kim@samsung.com (W.J.K.); 2 Department of Anesthesiology and Pain Medicine, Seoul St. Mary’s Hospital, College of Medicine, The Catholic University of Korea, Seoul 06591, Korea; huejung@catholic.ac.kr

**Keywords:** lumbosacral, pain, phase angle, radicular, transforaminal block

## Abstract

The mechanism of low back and leg pain involves mixed neuropathic and nociceptive components. Spinal neuropathic pain is related to increased levels of inflammatory cytokines and disrupted and increased permeability of the blood–spinal cord barrier, originally composed of tight junctions of capillary endothelial cells surrounded by lamina. The phase angle (PA) estimates cell membrane integrity using bioelectrical impedance analysis. We evaluated the predictive value of the PA for analgesic efficacy in lumbosacral transforaminal block. We retrospectively collected data from 120 patients receiving transforaminal blocks for lumbosacral radicular pain and assessed the PA before and 5 min following the block. Responders (group R) and non-responders (group N) were defined by ≥50% and <50% pain reduction, respectively, on a numerical rating scale, 30 min following the block; clinical data and the PA were compared. Among the 109 included patients, 50 (45.9%) and 59 (54.1%) had ≥50% and <50% pain reduction, respectively. In group N, the PA change ratio showed 88.1% specificity, 32.0% sensitivity, and 62.4% accuracy; a ratio of <0.087 at 5 min following the block predicted non-response. A PA change ratio of <0.087 at 5 min following lumbar transforaminal blocks predicted non-responders with high specificity.

## 1. Introduction

Lumbar epidural injection of local anesthetics and steroids is one of the methods of managing low back and radicular pain [1]. As one of several approaches, the transforaminal approach can deliver a small volume of injectate close to the site of pathology, presumably into a nerve root, to reduce inflammation and swelling [1,2,3,4,5]. However, objective metrics for assessing pain relief have not been well evaluated. Commonly used self-reported pain questionnaires are based on patient subjective responses, which may be affected by various factors, such as comorbid conditions and/or psychosocial causes [6,7,8]. Several objective pain tools, including skin conductance, nociception index, and pupillometry, have been reported; however, they involve high costs, limited availability, invasiveness, and/or various levels of accuracy [9]. In our pervious study on lumbar transforaminal blocks, we evaluated the correlation between perfusion index change and analgesic efficacy. We calculated the perfusion index change ratio from the perfusion index values before and 5 min after the block, and a ratio of >0.27 was found to be significantly related to pain relief; however, the perfusion index value was greatly changed by the assessing environment [10]. Early identification of the pain status is crucial for developing further treatment plans [6]. In cases where pain persists after the block, physicians consider further work-up to clearly diagnose and/or repeat blocks to relieve symptoms. Therefore, pain assessment after treatment is a cornerstone for decision-making regarding subsequent management. Pain monitoring by combining multiple autonomic signals (finger photoplethysmogram amplitude, skin conductance, heart rate, and heart rate variability) has shown limited correlation for detecting nociception [11]. Bioelectrical impedance analysis (BIA) is a technique used to measure body impedance by introducing a small current to the body; it is based on the principle that biological tissues act as conductors, semiconductors, or insulators [12,13]. The phase angle (PA) is a value derived from BIA [14]. The PA is estimated based on the ratio of reactance to resistance [12]. Resistance is the degree of obstruction of current flow caused by non-conductive tissue components, such as fat mass [12]. Reactance is an additional conduction delay, which is related to the capacitance of the cell membrane and tissue interfaces [12]. Reactance involves the integrity of cell membranes and the body cell mass [12]. The PA is positively associated with reactance and inversely associated with resistance [12]. In previous studies, a low PA has been observed in cases of a disrupted cellular membrane (malnutrition and inflammation) [15,16]. The PA may also predict poor survival outcomes in colorectal, lung, breast, or pancreatic cancers, leukemia, human immunodeficiency infection, and end-stage renal disease [17,18]. Recently, the PA showed diagnostic value in dysmobility syndromes characterized by impaired muscle function and performance status [12]. Lumbosacral radicular pain has mixed characteristics of neuropathic and nociceptive pain by spinal nerve roots inflammation and impingement [19,20,21]. We attempted to determine whether PA could reflect the pain status by measuring cell integrity before and after neural inflammation treatment. This study was performed to evaluate the predictive value of PA for analgesic efficacy in patients undergoing lumbosacral transforaminal blocks.

## 2. Materials and Methods

### 2.1. Patients

**Figure 1:**
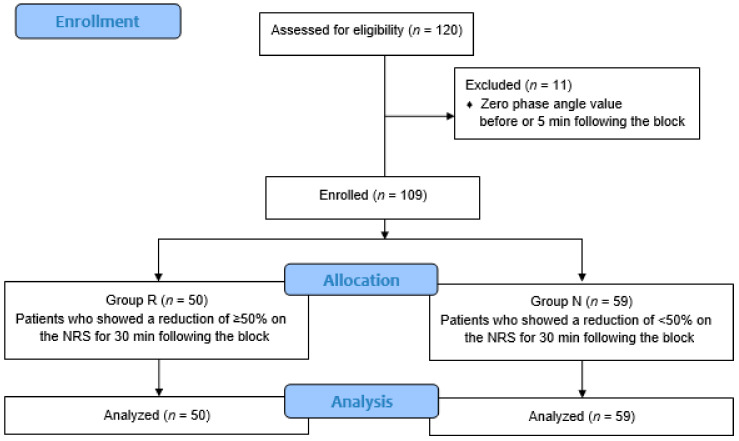
Flow diagram of the study. NRS: numerical rating scale.

We retrospectively reviewed the electronic medical records of patients with lower back and leg pain, who underwent a transforaminal block between February and September 2020 at a single tertiary care hospital; the patients were aged between 46 and 84 years. The inclusion criteria were as follows: (a) a primary diagnosis of lower back pain radiating to the lower limbs, (b) availability of a cross-sectional imaging study (either CT (computed tomography) or MRI (magnetic resonance imaging)) of the lumbosacral spine in patients diagnosed with spinal stenosis or herniated nucleus pulposus. This study was approved by our departmental ethics committee (SMC 2020-09-170) and registered with CRIS (Clinical Research Information Service of the Korea National Institute of Health, http://cris.nih.go.kr/cris/index.jsp, KCT0005642). The need for individual consent was waived by the institutional review board, as this was a retrospective study involving medical record review. Among the 120 patients assessed for eligibility, 11 were excluded due to zero PA values at T0 or T5, which led to zero PA change ratios on calculation; thus, the data of 109 patients were analyzed. We defined responders, or group R, as patients who showed a reduction of ≥50% on a numerical rating scale (NRS, ranging from 0 = no pain to 10 = absolutely intolerable pain) for pain at T30, and non-responders, or group N, as those who showed a reduction of less than 50% at T30 (Figure 1). The demographic and clinical data are summarized in Table 1.

### 2.2. Intervention

**Figure 2:**
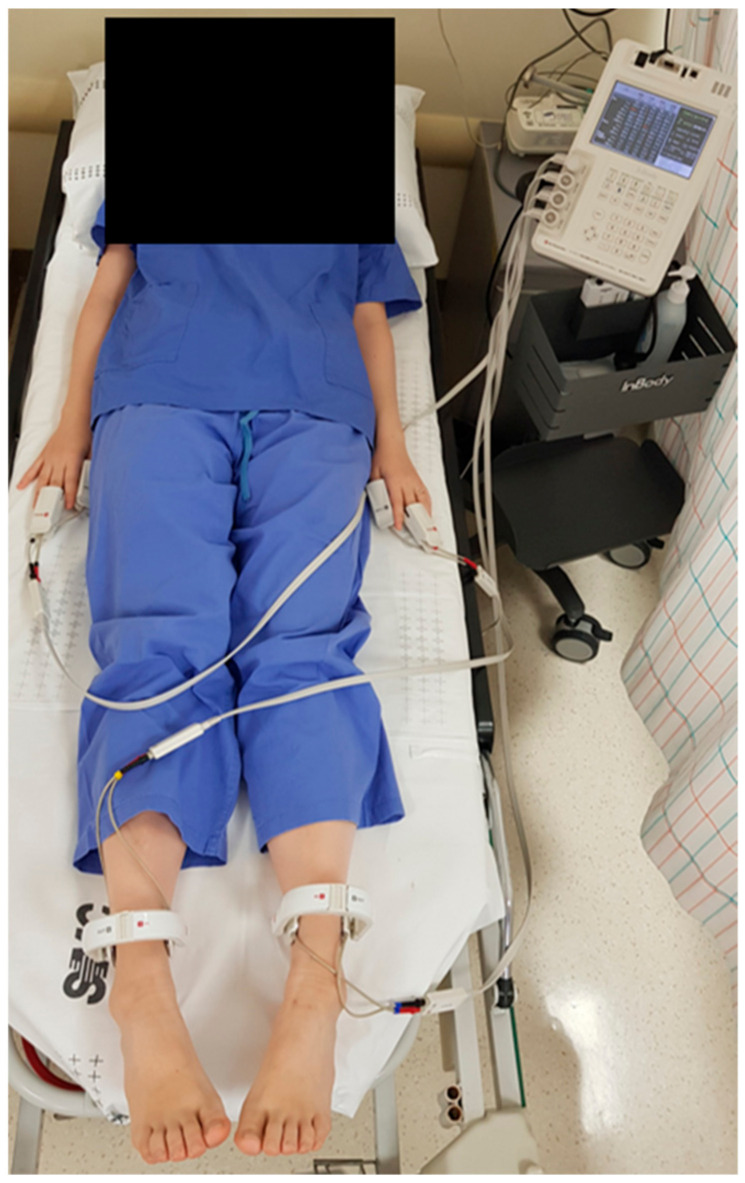
Whole-body bioelectrical impedance analysis measurement.

All procedures were performed under fluoroscopic guidance and were standardized. The lesion level for transforaminal injections was selected based on clinical manifestations, physical examination, and review of imaging studies. Lesion severity was categorized at any of three different levels (mild, moderate, or severe) by reviewing the imaging data. Exclusion criteria included any history of lumbosacral surgery, lumbosacral neuroplasty, neoplastic disease, or peripheral vascular disease. Patients were placed in the prone position, and anteroposterior and lateral view images were obtained using a C-arm (OEC series 9800, General Electronics, New York, NY, USA) to ensure proper site of entry. Following aseptic preparation and application of 1% lidocaine, a 23-gauge Tuohy needle (Tae-Chang Industrial Co., Seoul, Korea) was inserted into the skin surface over the upper quadrant of the target foramen. Aspirations were routinely performed for assessing the presence of blood or cerebrospinal fluid. When negative for aspirate, 0.5–2 mL of contrast medium (Omnipaque^®^, 300 mgI·mL^−1^, GE Healthcare, Little Chalfont, Buckinghamshire, UK) was injected to confirm that the point was well placed in the epidural space. After confirming that the contrast had spread throughout the epidural space, a total volume of 5 mL (containing 0.4% lidocaine, dexamethasone, hyaluronidase 750 IU, and normal saline) was infused. Following the procedure, patients were observed for any adverse effects. Whole-body BIA measurement was assessed using the Inbody S10 (Biospace Co., Ltd., Seoul, Korea). Following the standardized protocol, electrodes were placed on the bilateral hands and bare feet for 2 min [12] (Figure 2). The PA was calculated using the reactance and resistance database obtained at 50 Hz; we collected the PA value of the affected lower limb [12] prior to treatment (T0) and 5 min following transforaminal injection (T5). Temperature was assessed using a touch thermometer (IntelliVue MP70 patient monitor, Philips Healthcare, Best, the Netherlands) on the dorsum of the foot of the affected lower limb at T0 and T5. The room temperature was maintained at 23–25 °C. Pain and cold sensations of the affected lower limb were scored and recorded using a NRS at T0 and 30 min following transforaminal injection (T30).

### 2.3. Statistical Analysis

All data were analyzed using SAS 9.4 (SAS Institute, Cary, NC, USA). Data are expressed as the mean ± standard deviation (*SD*) or numbers (proportion), as appropriate. Demographic data for the two groups were compared using a Chi-square test, *t*-test, or Fisher’s exact test. To minimize individual variance in PA absolute values, we calculated the PA change ratio (PA at T5 − PA at T0/PA at T0). Temperature change (temperature at T5 − temperature at T0) was calculated. The PA change ratio was compared using the Wilcoxon rank sum test. The cut-off value of the PA change ratio was analyzed using Youden’s index. A *p*-value less than 0.05 was considered statistically significant.

## 3. Results

Age, sex, diagnosis, duration of pain, lesion level, injection level, injection side, and attempt number did not differ between the two groups (Table 1). Body mass index and lesion severity was different between the groups (*p* = 0.014, *p* = 0.038, respectively) (Table 1). The PA value, PA change, PA change ratio, and temperature change are presented in Table 2. The PA value was higher at T0 and T5 in group R, but no significant difference was observed between the groups (Table 2). PA change, PA change ratio, and temperature change were not different between the groups (Table 2). In group N, the cut-off value of the PA change ratio was <0.087 at T5; the area under the receiver operating characteristic curve was 0.521. The PA change ratio showed 32.0% sensitivity, 88.1% specificity, 62.4% accuracy, and Youden’s index of 0.201 (Table 3). Pain severity was different at T30 between the groups (*p* < 0.001) (Table 4). Cold sensation did not differ at T0 and T30 between the groups (Table 4). None of the cases showed any evidence of dural puncture or neurologic complications.

## 4. Discussion

In the present study, we found that a PA change ratio of <0.087 showed high specificity for identifying non-responders following transforaminal block. We speculate that low PA change ratios are attributable to lesser relief of inflammation and/or swelling of the affected nerve roots and are related to non-improvement of cellularity and non-response after epidural injections. Excessive nerve root swelling is a known risk factor for persistent or recurrent radiating pain after lumbar surgery [22]. It leads to nerve root compression with hyperalgesia, allodynia, and radiating pain [22]. Therefore, evaluation of non-responders after blocks is critical for deciding on further treatment. PA can be helpful as a simple, non-invasive, and easy-to-use pain tool when used along with a subjective pain questionnaire.

Lumbosacral radicular pain is caused by irritation or compression of the affected nerve root [23]. It is caused by chemically mediated inflammatory reactions, as well as by a direct mass effect on the nerve root [24]. The mechanism of low back and leg pain involves mixed components of neuropathic and nociceptive mechanisms [25]. Spinal neuropathic pain is related to increased levels of inflammatory cytokines and disrupted and increased permeability of the blood–spinal barrier, originally composed of tight junctions of capillary endothelial cell surrounded by lamina [26]. Dysfunction and breakage of the blood–spinal cord barrier is observed in several neurodegenerative disorders, peripheral neural injury-induced inflammatory pain, and chemotherapy-induced neuropathic pain [26,27]. In a rat sciatic nerve model, recruitment of inflammatory materials outside the nerve led to damage of the epineurium and depletion of epineural adipocytes [21,28]. Simultaneously, pro-nociceptive and pro-inflammatory mediators can enter the sciatic nerve through microlesions caused by neural damage [28]. Therefore, non-myelinating Schwann cells may no longer protect non-myelinated neurons, causing tactile allodynia and loss of function of thermal sensitivity [28]. Transforaminal block is a valid procedure for the diagnosis and treatment of lumbosacral radicular pain, and acts by reducing inflammation and edema [29]. Steroid and local anesthetics through the foramen dilute inflammatory cytokines, reduce adhesion, improve blood circulation, suppress affected neural ectopic discharge, and reduce central sensitization [30]. We attempted to quantify pain changes using an objective tool after resolving nerve root inflammation with transforaminal block. Although the perfusion index was associated with pain relief following transforaminal block, perfusion index measurement is considerably sensitive to movement, and it fluctuates rapidly in response to surgical or other noxious stimuli, temperature, and stress, leading to changes in the perfusion index [31,32]. The PA is a parameter of BIA measurement, which reflects balance between cell hydration and body mass [33]. A low PA value is associated with cellular membrane damage and cell death, and a high PA value is correlated with proper cell membrane or cell function [33]. The PA depends on tissue features, cell size, cell function, and cellularity. Moreover, the PA is known as a cellular health indicator, but the prognostic value of PA may differ in various clinical situations [33,34].

In this study, we aimed to evaluate whether the PA has predictive value for analgesic efficacy after lumbosacral transforaminal blocks. There was no significant difference in the PA values between the groups. However, the PA change ratio showed 88.1% specificity in non-responders. The cut-off value of the PA change ratio was <0.087 at 5 min following the block. We followed up the PA at 5 min after the block because the perfusion index change ratio was significant at that time point, and it correlated with pain relief [10]. We suspect that the PA evaluation time was inadequate for reflecting improvement of inflammation and cellular integrity of the affected nerve roots after transforaminal blocks. Additionally, we included patients with different pain durations (acute and chronic pain), which may affect the functional dysmobility and inflammatory status, leading to bias regarding the PA value. Further studies will be needed to evaluate appropriate measurement times and to compare PA values between acute and chronic pain conditions. This study has several limitations. First, we categorized patients only using a numerical rating scale; we did not record the functional disability status. Second, the follow-up period of 30 min for detecting a response was inadequate for evaluating block efficacy. Third, we did not measure the nutrition status and pro-inflammatory or inflammatory markers in each patient; CRP (C-reactive protein) and TNF-α (tumor necrosis factor) are known to be related with cell plasma membrane instability [35,36]. Fourth, we did not enroll a sham group to compare with the PA values of patients who did not receive blocks. Fifth, the sample was heterogeneous in terms of age, ranging from 46 to 84 years, and we did not perform age adjustment because there was no significant difference in age between the groups. Finally, the sample size was small, but the power was 82.9%.

## 5. Conclusions

We observed that the PA change ratio has high specificity in identifying non-responders after lumbar transforaminal blocks. Further prospective controlled studies are needed to determine whether the PA provides superior diagnostic value in other forms of acute and chronic pain treatment.

## Figures and Tables

**Table 1:** Demographic and clinical characteristics.

Title	All Patients (*n* = 109)	Group R (*n* = 50)	Group N (*n* = 59)	*p*-Value
Age (year)	69.3 ± 8.0	69.2 ± 8.1	69.4 ± 7.9	0.895
Sex (male/female)	65/44	31/19	34/25	0.643
Body mass index (kg/m^2^)	25.8 ± 3.2	26.7 ± 3.5	25.1 ± 2.8	0.014
Diagnosis				1.000
Spinal stenosis	98 (89.9%)	45 (90.0%)	53 (89.8%)	
HNP	11 (10.1%)	5 (10.0%)	6 (10.2%)	
Duration of pain (month)				0.340
<3	4 (3.7%)	3 (6.0%)	1 (1.7%)	
3–12	21 (19.3%)	11 (22.0%)	10 (16.9%)	
>12	84 (77.1%)	36 (72.0%)	48 (81.4%)	
Lesion level				0.639
L2–3	1 (0.9%)	1 (2.0%)	0 (0.0%)	
L3–4	23 (21.1%)	11 (22.0%)	12 (20.3%)	
L4–5	64 (58.7%)	27 (54.0%)	37 (62.7%)	
L5–S1	21 (19.3%)	11 (22.0%)	10 (17.0%)	
Lesion severity				0.038
Mild	0 (0.0%)	0 (0.0%)	0 (0.0%)	
Moderate	66 (60.6%)	25 (50.0%)	41 (69.5%)	
Severe	43 (39.4%)	25 (50.0%)	18 (30.5%)	
Injection level				0.181
L2	1 (0.9%)	0 (0.0%)	1 (1.7%)	
L3	16 (14.7%)	10 (20.0%)	6 (10.2%)	
L4	47 (43.1%)	17 (34.0%)	30 (50.8%)	
L5	41 (37.6%)	20 (40.0%)	21 (35.6%)	
S1	4 (3.7%)	3 (6.0%)	1 (1.7%)	
Injection side				0.301
Left/Right	56/53	23/27	33/26	
Attempt number				0.248
1/2	106/3	50/0	56/3	

All data are presented as the mean ± *SD* or number (%) of patients. HNP: herniated nucleus pulposus; Group R: patients who showed a reduction of ≥50% on the numerical rating scale for pain 30 min following the block; Group N: patients who showed a reduction less than 50%; *p*-value < 0.05 was considered statistically significant.

**Table 2:** Phase angle and temperature.

	All Patients (*n* = 109)	Group R (*n* = 50)	Group N (*n* = 59)	*p*-Value
Phase angle value				
T0	6.42 ± 8.3	7.63 ± 12.0	5.40 ± 1.5	0.944
T5	6.64 ± 9.2	8.09 ± 13.4	5.42 ± 1.6	0.733
Phase angle change	0.22 ± 1.8	0.46 ± 2.3	0.02 ± 1.3	0.594
Phase angle change ratio	0.05 ± 0.4	0.09 ± 0.6	0.02 ± 0.3	0.713
Temperature change	−0.08 ± 0.2	−0.08 ± 0.2	−0.07 ± 0.2	0.933

All data are presented as the mean ± *SD*. T0: before treatment; T5: 5 min following the block; Phase angle change (phase angle at T5 − phase angle at T0); Phase angle change ratio (phase angle at T5 − phase angle at T0/phase angle at T0); Temperature change (temperature at T5 − temperature at T0); Group R: patients who showed a reduction of ≥50% on the numerical rating scale for pain 30 min following the block; Group N: patients who showed a reduction less than 50%; *p*-value < 0.05 was considered statistically significant.

**Table 3:** Diagnostic assessment of the phase angle change ratio in non-responders.

	Group N (*n* = 59)
AUROC	0.521 (95% CI 0.408–0.633)
Cut-off value	0.087
Sensitivity	32.0% (95% CI 0.208–0.458)
Specificity	88.1% (95% CI 0.775–0.941)
Accuracy	62.4% (95% CI 0.530–0.709)
Youden’s index	0.201

AUROC: area under the receiver operating characteristic curve; CI: confidence interval; Group N: patients who showed a reduction less than 50%.

**Table 4:** Pain severity and cold sensation over time.

	All Patients (*n* = 109)	Group R (*n* = 50)	Group N (*n* = 59)	*p*-Value
Pain severity (NRS)				
T0	6.48 ± 2.0	6.72 ± 2.2	6.27 ± 1.9	0.346
T30	3.66 ± 2.4	1.70 ± 1.7	5.32 ± 1.4	<0.001
Cold sensation (NRS)				
T0	2.06 ± 3.0	1.66 ± 3.0	2.39 ± 3.0	0.122
T30	1.26 ± 2.2	0.92 ± 2.0	1.54 ± 2.3	0.094

All data are presented as the mean ± *SD*. T0: before treatment; T30: 30 min following the block; NRS: numerical rating scale; Group R: patients who showed a reduction of ≥50% on the numerical rating scale for pain 30 min following the block; Group N: patients who showed a reduction less than 50%; *p*-value < 0.05 was considered statistically significant.

## Data Availability

The data presented in this study are available on request from the corresponding author. The data are not publicly available due to ethical reason.
